# Current News

**Published:** 1894-04

**Authors:** 


### Current News.

### DENTAL SOCIETY OF THE STATE OF NEW YORK.

The Twenty-sixth Annual Meeting of the above Society will be held in the Y. M. C. A. Building, Albany, on the 9th and 10th of May next, at which time essays will be presented by S. H. Guilford, D.D.S., Ph.G., of Philadelphia, on "Crown- and Bridge-Work: A Protest against Some of its Abuses;" J. Allen Osmun, M.D.S., of

Newark, N. J., "Can Pyorrhœa be cured?" W. J. Turner, M.D., D.D.S., of Brooklyn, "The Use of Peroxide of Sodium as recommended by Dr. E. C. Kirk;" R. M. Sanger, D.D.S., of East Orange, N. J.

A cordial invitation is extended to the profession.

F. T. Van Woert, Brooklyn, N. Y.,
*President.*

C. S. Butler, Buffalo, N. Y.,
*Secretary.*

---

### ANNUAL MEETING OF HARVARD ODONTOLOGICAL SOCIETY.

The Sixteenth Annual Meeting of the Harvard Odontological Society was held at Young's Hotel, Boston, Saturday, February 24, at 5.30 P.M., the President, Dr. Forrest G. Eddy, in the chair.

The Recording and Corresponding Secretaries, Treasurer, and Editor read their annual reports, which were accepted. The Recording Secretary stated that during the year twelve papers had been read before the Society, and that at every meeting save two, members had presented for inspection specimens or models in the line of dentistry.

Among the guests present at the dinner were Hon. George A. Marden, Lowell, Mass.; Hon. Charles H. George, postmaster, Providence, R. I.; Rev. Charles G. Ames, Boston; Louis A. Brandeis, LL.B., Boston; Hon. William F. Sawyer, president Massachusetts College of Pharmacy; Rev. Alexander McKenzie, D.D., Cambridge, Mass.; Hon. Henry H. Sprague, Boston; Rev. F. W. Merrick, Boston; G. A. Leland, M.D., Boston. His Excellency, Governor Greenhalge, was unable to be present, but the State was represented by Adjutant-General Dalton.

The orator of the evening was George F. Grant, D.M.D., Boston, who spoke on "The Development of Our Profession." He contrasted dentistry of to-day with that of twenty-five years ago, and showed that, although it had attained marvellous growth, it still lacked development, there being numberless channels, at present comparatively unexplored, in which the investigator could work. He said that dentistry as a profession is unattractive to the world at large. Most professions have access to the public through the magazines of the day, but ours has not; it is not advertised in that way. What we most need is to attract to our ranks men of worth; we should get in the "best blood."

The speeches of the guests were entertaining and full of encouragement.

The following-named officers were elected for the ensuing year: President, Forrest G. Eddy, D.M.D., Providence, R. I.; Recording Secretary, Waldo E. Boardman, D.M.D., Boston; Corresponding Secretary, James Shepherd, D.M.D., Boston; Treasurer, Dwight M. Clapp, D.M.D., Boston; Editor, Henry L. Upham, D.M.D., Boston.

*Executive Committee.*—Waldo E. Boardman, D.M.D., Boston; Jere. E. Stanton, M.D., D.M.D, Boston; A. H. Stoddard, D.M.D., Boston.

W. P. Cooke, D.M.D., Boston, was elected orator for 1895.

James Shepherd,
*Corresponding Secretary.*

---

### THE TRANSACTIONS OF THE WORLD'S COLUMBIAN DENTAL CONGRESS.

The Transactions are now nearly ready for the final proof-reading; it is therefore very desirable that those who want extra copies, and those who would like to subscribe for them, do so at once, as it is the intention of the committee of publication to print only a sufficient number of copies to supply the members of the Congress and those who have subscribed for them. The subscription price is ten dollars.

Subscriptions should be sent to Dr. John S. Marshall, Treasurer, 1003 Venetian Building, Chicago, Ill.

---

### CENTRAL DENTAL ASSOCIATION OF NORTHERN NEW JERSEY.

At the annual meeting of the Central Dental Association of Northern New Jersey, held in Newark on the evening of February 19, 1894, the following officers were elected for the ensuing year:

President, Dr. Thomas Moore, Paterson; Vice-President, Dr. William P. Richards, Orange; Secretary, Dr. William L. Fish, Newark; Treasurer, Dr. Charles A. Meeker.

*Executive Committee.*—Dr. Geo. E. Adams, South Orange; Dr. Oscar Adelberg, Elizabeth; Dr. Fred. A. Barlow, Jersey City; Dr. Walter Woolsey, Elizabeth; Dr. William E. Linsted, New Brunswick.

William L. Fish,
*Secretary.*

